# An extremely atypical presentation of esophageal squamous cell carcinoma with pancreatic and hepatic metastases: A case report and overview of the literature

**DOI:** 10.1097/MD.0000000000025785

**Published:** 2021-05-21

**Authors:** Lei Zhang, Xin Long, Zheng-Nan Hu, Yu Wu, Jia Song, Bi-Xiang Zhang, Wei-Xun Chen

**Affiliations:** Hepatic Surgery Center, Department of Surgery, Tongji Hospital, Tongji Medical College, Huazhong University of Science and Technology, Wuhan, China.

**Keywords:** esophageal carcinoma, esophageal squamous cell carcinoma, pancreatic metastasis, primary pancreatic carcinoma

## Abstract

**Rationale:**

Esophageal carcinoma is an aggressive cancer with extremely poor therapeutic outcomes due to its high metastatic potential and a significant risk of recurrence after radical resection. Liver is the most common metastatic target organ of esophageal carcinoma, followed by the lungs, bones, and brain. Few cases of solitary pancreatic and hepatic metastases of esophageal carcinoma have been reported.

**Patient concerns:**

We report the case of a 67-year-old male presenting with pancreatic and hepatic lesions. In addition, a friable lesion with an irregular nodular surface in the distal esophagus was detected by esophagogastroduodenoscopy.

**Diagnosis:**

Pathohistological examination confirmed esophageal squamous cell carcinoma. The pancreatic lesion was also biopsied via ultrasound-guided fine needle aspiration, which also revealed squamous cell carcinoma. The hepatic lesion was also identified as metastatic carcinoma by magnetic resonance imaging, most likely of the same origin.

**Interventions:**

Due to comorbidities that precluded surgery, the patient was administered adjuvant therapy and a multidisciplinary decision was made for palliative care.

**Outcomes:**

The patient died 1 month later due to multiorgan failure caused by hemorrhage from a peptic ulcer.

**Conclusion:**

To our knowledge, this is only the sixth case of pancreatic metastasis of esophageal squamous cell carcinoma. This case report suggests to clinicians the importance of considering potential comorbidities in every patient with advanced cancer, such as gastric ulcer and cachexia.

## 1 Introduction

Esophageal cancer is the eighth most common and sixth most lethal malignancy worldwide, with 400,200 deaths.^[1]^ The prognosis for esophageal cancer with distant organ metastases remains dismal and its treatment poses a challenge for oncologists.^[2]^ In spite of recent advances in multimodal treatment using esophagectomy and definitive chemo-radiotherapy or chemotherapy, patients with esophageal cancer have poor outcomes due to this cancer's propensity for metastasis.^[3]^ Pancreatic metastatic malignancies have been observed in up to 4.9% of cases for some cancers.^[4–8]^ However, pancreatic metastasis from esophageal cancer is rare, with frequencies of 0.1%, 2.9%, and 0.7%, for esophageal cancer, esophageal squamous cell carcinoma (ESCC), and metastatic esophageal cancer, respectively.^[9]^ According to pathological examination of autopsy samples, another study reported that the rate of esophageal carcinoma with pancreatic metastasis is as high as 3.9%.^[10]^ To the best of our knowledge, there are only few reports in the English literature of ESCC with pancreatic metastasis. Here, we describe a case of ESCC with simultaneous pancreatic and hepatic metastases, followed by an overview of the relevant literature.

## 2 Case report

**Figure 1:**
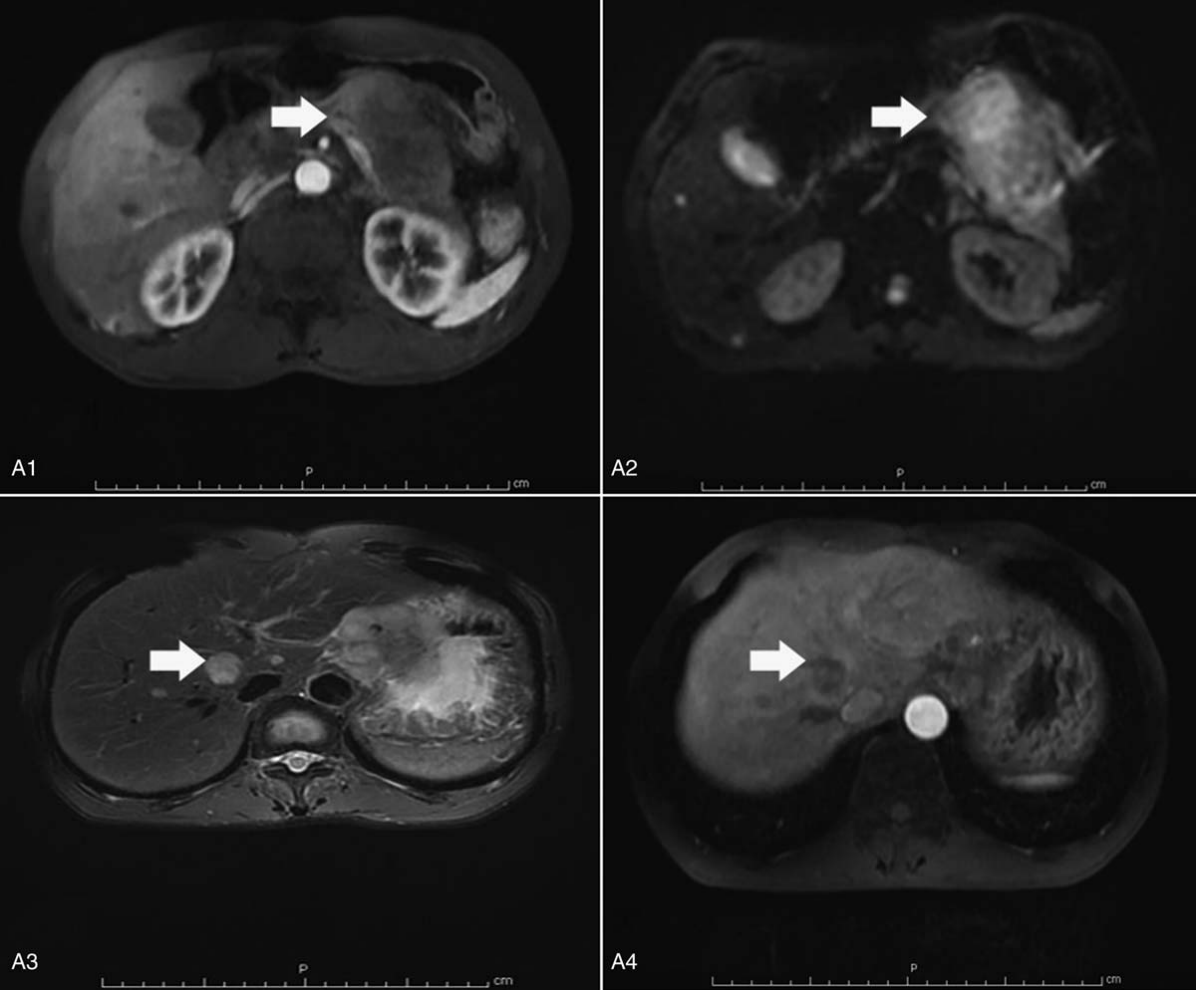
Radiological examination of the reported case. Abdominal MRI scanning showed a hypoattenuating lesion located in the body and tail of the pancreas, invading the spleen vein. MRI = magnetic resonance imaging.

**Figure 2:**
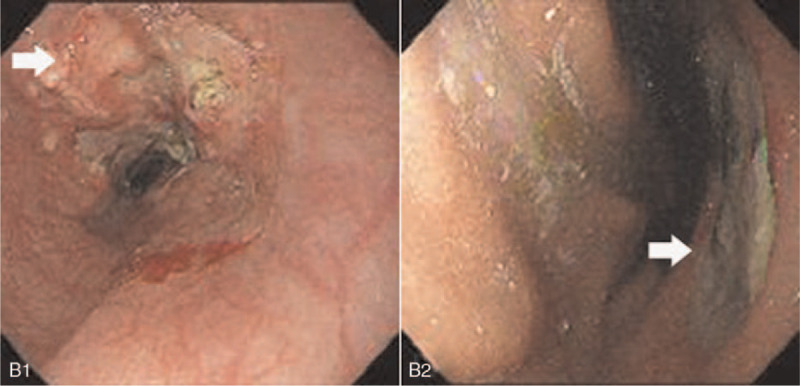
Esophagogastroduodenoscopy finding shows a friable erythematous lesion with an irregular nodular surface extending between 33 and 36 cm from the incisors and a large ulcer in the gastric mucosa located on the lesser curvature of the upper gastric body.

**Figure 3:**
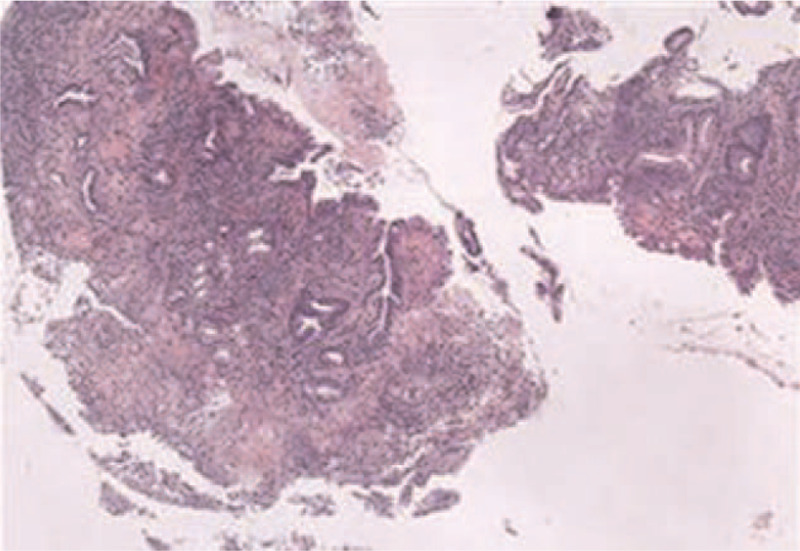
The pathology from the esophageal lesion of the reported case.

**Figure 4:**
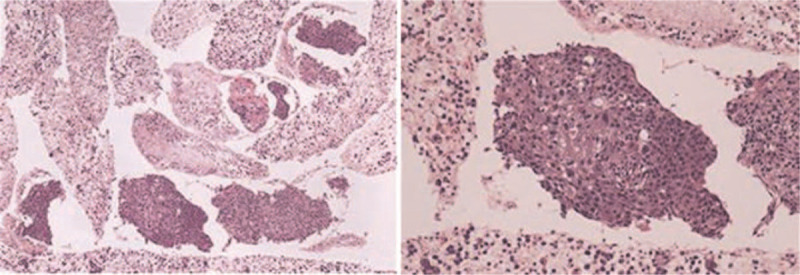
Cytology examination and immunohistochemical stains of the pancreatic mass.

A 67-year-old male was referred to our hospital following a diagnosis of pancreatic and hepatic lesions which were identified by computed tomography. He had felt pain in the upper right abdomen for 1 month, worsening for 1 week. He complained of pain after eating, radiating to the back, along with intermittent nausea and vomiting. He had a weight loss of 5 kg over the preceding 2 months. Initial physical examination revealed normal vital signs. The results of laboratory tests were almost in the normal range, as well as tumor biomarkers including alpha-fetoprotein of 2.6 ng/mL (reference range 0–9 ng/mL), carbohydrate antigen 19-9 of 23 U/mL (normal value: <37 U/mL), carbohydrate antigen 125 of 17 U/mL (normal value: <35 U/mL), and carcinoembryonic antigen of 5.57 ng/mL (normal value: <4.1). Additional tests for hepatitis B showed hepatitis B e antigen (–) and antihepatitis B e antibodies (+), while no hepatitis B virus was detected by real-time polymerase chain reaction. Magnetic resonance imaging (MRI) of the abdomen revealed a 5.1 cm × 6.9 cm mass in the body and tail of the pancreas invading the spleen vein, with multiple well-defined low-attenuation areas scattered throughout the lesion (Fig. 1A1). On magnetic resonance (MR) diffusion-weighted imaging, the mass exhibited hyperintensity with restricted diffusion (Fig. 1A2). T2-weighted MR images revealed multiple hyperintense nodules in the liver with their largest diameter being 2.3 cm, while MR perfusion-weighted imaging showed enhancement on dynamic enhancing study, suggestive of metastatic disease (Fig. 1A3 and A4). The patient underwent an esophagogastroduodenoscopy with endoscopic ultrasound (EUS) to evaluate the patient's dysphagia and weight loss. This revealed a friable erythematous lesion with an irregular nodular surface surrounding the esophagus and extending between 33 and 36 cm from the incisors (Fig. 2B1). The esophagogastroduodenoscopy also revealed a large ulcer in the gastric mucosa located on the lesser curvature of the upper gastric body (Fig. 2B2). A pathohistological examination of the esophageal lesion confirmed squamous cell carcinoma (Fig. 3). Therefore, the patient was initially diagnosed with ESCC with pancreatic and hepatic metastases. Ultrasound-guided fine-needle aspiration (FNA) was performed on the body and tail of the pancreas. Cytological examination of the pancreatic mass was highlighted by immunohistochemical staining for PCK, P63, P40, and CK7, and it was presumed to be derived from primary ESCC (Fig. 4). The combined positive score of the programmed cell death-ligand 1 (PD-L1) expression level was <1%. Whole-exome sequencing was applied to the tissue extracted during ultrasound-guided FNA, and the data were used to determine the presence of nonsynonymous mutations, as well as the status of tumor mutation burden (TMB) and microsatellite instability (MSI) by bioinformatics methods. The TMB was determined to be 5.26 mutations/Mb, and a total of 20 nonsynonymous mutations were detected in the whole genome, including 6 insertion-deletion mutations, 9 single-nucleotide variants, and 5 copy number variations. The tumor harbored clinically relevant mutations in *CDKN2A*, *CCND1*, and *FGF19.* Only *NR-24* contained an single-nucleotide variants, suggesting MSI-low (MSI-L). The patient was administered adjuvant therapy with erlotinib plus gemcitabine recommend by the Food and Drug Administration and a multidisciplinary decision was made for palliative care given his comorbidities and multiple sites of metastases from his esophageal squamous cell cancer. Unfortunately, the patient died 1 month later due to multiorgan failure caused by hemorrhage from a stomach ulcer.

## 3 Discussion

Pancreatic tumors can be either of endocrine or exocrine origin. Exocrine tumors are derived from acinar and ductal cell, while endocrine tumors are derived from islet cells.^[11,12]^ Most pancreatic tumors are primary, with adenocarcinoma accounting for about 80%. Additional malignant tumors of the pancreas include small cell carcinoma, squamous cell carcinoma, metastatic lesions, and acinar cell carcinoma.^[13]^ Malignancies that have been reported to metastasize to the pancreas include liver, breast, lung, colorectal, and renal cancers, as well as melanoma. These metastases account for about 2% of pancreatic cancer cases. When metastases occur, there is no specific preference for any region of the pancreas.^[14]^ There may be slight differences in the appearance upon imaging using methods such as computed tomography and MRI, whereby primary adenocarcinoma may show hypoenhancement as opposed to peripheral or homogeneous contrast enhancement in metastases. In addition, EUS of pancreatic metastases shows more well-defined margins compared to primary adenocarcinoma. A tissue biopsy should be made before surgery using EUS-guided FNA to distinguish a solitary pancreatic metastasis from a primary cancer and a double or triple primary pancreatic cancer. Because the normal pancreas does not contain squamous epithelium, only 0.7% of pancreatic cancer cases are primary squamous cell carcinoma of the pancreas.^[5]^ Hence, squamous cells detected upon sampling a pancreatic lesion represent a very unusual and unanticipated discovery. When identified, metastasis from another site should be excluded, and the astute clinician should focus on uncovering the primary malignancy of squamous cell origin.^[15]^

To the best of our knowledge, only 5 cases of pancreatic metastasis of ESCC have been reported in the English literature to date (Table 1). Esfehani et al^[16]^ reported a case of metachronous pancreatic metastasis of ESCC in a 59-year old woman who had undergone transthoracic esophagectomy and cervical esophago-gastrostomy with adjuvant chemo-radiotherapy 4 years earlier. Distal pancreatectomy, splenectomy, and adjuvant chemotherapy were performed for the pancreatic metastasis with splenic vessel invasion, and there was no recurrence within the 6-month follow-up. Park et al^[15]^ reported the case of a 58-year-old male with primary hepatocellular carcinoma and pancreatic metastasis from esophageal cancer. He had undergone esophagectomy with distal pancreatectomy and radiofrequency ablation for hepatocellular carcinoma. Histologically, the partial esophagectomy and distal pancreatectomy specimens presented as squamous cell carcinoma. The patient had received 5-fluorouracil/cisplatin combination therapy 6 times after the surgery. Sawada et al^[17]^ reported that a 73-year-old man developed hepatic portal venous embolism caused by gastric wall infarction due to the expansion of an isolated pancreatic metastasis from ESCC. Hiroshi et al^[18]^ reported the case of a 68-year-old male with metachronous pancreatic metastasis that was resected 2 years after salvage esophagectomy for local recurrence of ESCC followed by reconstruction with a conduit colon graft via a subcutaneous route. There was no recurrence 9 months after surgical resection of the pancreatic metastasis. Wataru et al^[19]^ reported the case of a 70-year-old female with metachronous pancreatic metastasis who underwent distal pancreatectomy 11 years after transthoracic radical esophagectomy for ESCC. A previous report demonstrated that resection of the pancreatic metastasis with no widespread disease in 16 patients with different primary tumors led to longer overall and disease-free survival^[20]^ Surgical resection may be reasonable for treatment and obtaining a definite diagnosis, as well as for improving patient survival. In our case, the cytological examination by ultrasound-guided FNA revealed that the pancreatic mass was squamous cell carcinoma according to both morphologic features and immunohistochemical analysis. However, metastatic disease may show peripheral or homogeneous contrast enhancement on abdominal MRI instead of multiple well-defined low-attenuation areas scattered throughout the lesion. Considering the MRI characteristics, synchronous double primary cancers of the pancreas and the esophagus were also possible, even if such cases are very rare.

**Table 1:** Reported cases of pancreatic metastasis from oesophageal carcinoma.

Authors (year of publication)	Patient age (yr)	Sex	Synchronous/metachronous	Interval between metastases (mo)	Surgery	Adjuvant therapy	Follow-up outcomes (mo)
Esfehani et al (2011)	59	F	Metachronous	48	DP	5-FU	4 alive
Park et al (2013)	58	M	Synchronous	0	DP	FP	6 alive
Sawada et al (2013)	73	M	Synchronous	0	NA	NA	3 dead
Hiroshi et al (2014)	68	M	Metachronous	24	DP	FP	9 alive
Wataru et al (2019)	70	F	Metachronous	132	DP	NA	24 alive

Most pancreatic carcinomas contain differentiated squamous cells, which makes them a type of adenosquamous carcinoma. Therefore, the World Health Organization does not classify the adenosquamous carcinoma of the pancreas as a distinct entity. Five theories have been proposed to explain the development of pancreatic squamous cell carcinoma.^[21]^

(1) Squamous transformation of a pre-existing adenocarcinoma;
(2) malignant transformation of a primitive cell capable of differentiating into either squamous or glandular carcinoma;
(3) malignant transformation of an aberrant squamous cell;
(4) malignant transformation of a squamous metaplasia of the ductal epithelium;
(5) tumor collision.

To the best of our knowledge, only 7 cases of double primary carcinoma of the pancreas and esophagus have been reported in the literature (Table 2). Kurosaki I et al^[22]^ reported the case of a patient who underwent a thoracic esophagectomy and pylorus-preserving pancreatoduodenectomy in a 1-stage procedure for asynchronous primary cancer of the thoracic esophagus and a primary intraductal papillary tumor of pancreas. Histologically, the esophageal tumor presented as squamous cell carcinoma, while the pancreatic lesion contained mucus and an intraductal polypoid tumor with ecstatic pancreatic ducts. Shin et al^[23]^ evaluated the clinicopathological features of 113 patients with pancreatic ductal adenocarcinoma and double primary tumors. The stomach was the most common location for a double primary tumor, affecting 26 patients, while only 1 case had a second primary tumor of the esophagus (squamous cell carcinoma) in addition to the pancreas. According to the literature, patients who underwent resection of the pancreatic cancer before the diagnosis of metachronous tumors had better overall survival than those resected after the diagnosis of metachronous tumors or those resected synchronously. Kim et al^[24]^ reported the case of a 65-year-old male who underwent combined pancreaticoduodenectomy and transhiatal esophagectomy. Final pathology revealed a mucinous adenocarcinoma of the pancreas and a poorly differentiated adenocarcinoma in the esophagus infiltrating into the muscularis propria. Gyorki et al^[25]^ described a case of a neuroendocrine tumor of the pancreatic head and an adenocarcinoma of the distal esophagus diagnosed synchronously but successfully managed metachronously. The patient underwent an esophagectomy with a colonic reconstruction, followed some months later by pylorus-preserving pancreaticoduodenectomy. Banerjee et al^[26]^ reported a 56-year-old male with synchronous advanced adenocarcinomas of the pancreatic body and gastro-esophageal junction. The patient underwent a combination of esophagectomy with Appleby procedure and adjuvant chemotherapy. Satiya et al^[27]^ reported the case of a 54-year-old male with synchronous cancers of the pancreas and gastroesophageal junction. The histopathological investigation of the pancreatic lesion confirmed adenocarcinoma and the gastroesophageal junction nodule proved to be adenocarcinoma with mucinous features. Ozawa et al^[28]^ reported a case of synchronous moderately differentiated ESCC and a well-differentiated adenocarcinoma of the pancreatic head. To reduce the surgical invasiveness and avoid serious postoperative complications, the patient underwent a 2-stage operation that included a thoracoscopic esophagectomy, a percutaneous endoscopic gastrostomy, and a pancreaticoduodenectomy.

**Table 2:** Reported cases of double primary of the pancreas and esophagus.

Authors (year of publication)	Patient age (yr)	Sex	Synchronous/metachronous	Surgery	Adjuvant therapy	Follow-up (mo)
Kurosaki I et al (2000)	72	M	Synchronous	TE + PPPD	radiotherapy	60
Yul Kim et al (2011)	68	M	Synchronous	TE + PD	NA	NA
DE Gyorki et al (2011)	58	M	Synchronous	TE, PPPD (metachronous resection)	NA	NA
Banerjee JK et al (2018)	56	M	Synchronous	TE + Appleby procedure	Capecitabine + oxaliplatin + gemcitabine	NA
Satiya J et al (2019)	54	M	Synchronous	NA	FOLFIRINOX	6
Ozawa H et al (2020)	70	F	Synchronous	VATS, PD (metachronous resection)	NA	12

In our case, surgical resection for treatment and obtaining a definite diagnosis was excluded due to few guidelines for pancreatic and hepatic metastasis from ESCC or triple primary carcinoma. On the basis of a review of the literature, we inferred that pancreatic metastasectomy may be a useful treatment option for isolated pancreatic metastasis from ESCC. Early diagnosis of multiple primary carcinomas is crucial for improved patient outcomes. Moreover, established guidelines for treating primary cancer with double metastatic lesions are needed.

## Author contributions

**Data curation:** Lei Zhang, Xin Long, Zheng-Nan Hu.

**Formal analysis:** Lei Zhang, Zheng-Nan Hu.

**Investigation:** Lei Zhang, Yu Wu.

**Methodology:** Yu Wu.

**Resources:** Jia Song.

**Software:** Lei Zhang, Jia Song.

**Supervision:** Wei-Xun Chen.

**Validation:** Bi-Xiang Zhang, Wei-Xun Chen.

**Visualization:** Bi-Xiang Zhang, Wei-Xun Chen.

**Writing – original draft:** Lei Zhang.

**Writing – review & editing:** Lei Zhang, Bi-Xiang Zhang, Wei-Xun Chen.
